# Tailor-Made Specific Recognition of Cyromazine Pesticide Integrated in a Potentiometric Strip Cell for Environmental and Food Analysis

**DOI:** 10.3390/polym11091526

**Published:** 2019-09-19

**Authors:** Nashwa S. Abdalla, Abd El-Galil E. Amr, Aliaa S. M. El-Tantawy, Mohamed A. Al-Omar, Ayman H. Kamel, Nagy M. Khalifa

**Affiliations:** 1 Department of Chemistry, Faculty of Science, Ain Shams University, Abbasia 11566, Cairo, Egypt; 2 Pharmaceutical Chemistry Department, Drug Exploration & Development Chair (DEDC), College of Pharmacy, King Saud University, Riyadh 11451, Saudi Arabia; 3 Applied Organic Chemistry Department, National Research Center, Dokki 12622, Giza, Egypt; 4 Department of Pharmaceutical Chemistry, National Organization for Drug Control and Research, Giza 22311, Egypt

**Keywords:** cyromazine (CR), solid-contact ISEs, screen-printed, molecularly imprinted polymers (MIPs), polyaniline (PANI)

## Abstract

Screen-printed ion-selective electrodes were designed and characterized for the assessment of cyromazine (CYR) pesticide. A novel approach is to design tailor-made specific recognition sites in polymeric membranes using molecularly imprinted polymers for cyromazine (CR) determination (sensor I). Another sensor (sensor II) is the plasticized PVC membrane incorporating cyromazine/tetraphenyl borate ion association complex. The charge-transfer resistance and water layer reached its minimal by incorporating Polyaniline (PANI) solid-contact ISE. The designed electrodes demonstrated Nernstain response over a linear range 1.0 × 10^−2^–5.2 × 10^−6^ and 1.0 × 10^−2^–5.7 × 10^−5^ M with a detection limit 2.2 × 10^−6^ and 8.1 × 10^−6^ M for sensors I and II, respectively. The obtained slopes were 28.1 ± 2.1 (r^2^ = 0.9999) and 36.4 ± 1.6 (r^2^ = 0.9991) mV/decade, respectively. The results showed that the proposed electrodes have a fast and stable response, good reproducibility, and applicability for direct measurement of CYR content in commercial pesticide preparations and soil samples sprayed with CYR pesticide. The results obtained from the proposed method are fairly in accordance with those using the standard official method.

## 1. Introduction

Pesticides are agrochemicals used in pests controlling and to increase product quality and yield. Excessive use of pesticides leads to threats to public health and food safety as well as insect resistance to these pesticides and environmental pollution [1]. Cyromazine (CYR) is a triazine derivative and is developed as an insecticide. It can be used for controlling the growth of insects and also as an acaricide, with contact action that interferes with moulting and pupation [2]. When it is added to animal food, it prevents hatching of fly larvae on sheep and lambs. So, it has an interesting application in saving animal yield. It is also used against houseflies and mosquitoes [3,4]. Cyromazine (CYR) is being also applied in massive amounts to save agricultural crop yields. Celery, spinach, and lettuce are top considering the percentage of crop [5]. It is also applied for fly control in mushrooms and vegetables [6]. Cyromazine is slightly toxic by ingestion, with reported oral LD_50_ values of 3387 mg^−1^ kg in rats [7]. It was noticed that CRY has non-noticeable toxicity in humans and animals. Despite that, recent toxicological studies reported that cyromazine can produce mammary tumors in rats; as its mammary tumor-producing analogues [8,9]. Photocatalytic degradation of CYR under normal environmental conditions produces the carcinogenic metabolite melamine [10,11]. In 2008, melamine was used in adulteration of milk to increase its protein value because it is rich in nitrogen content. Melamine causes renal failure and associated deaths in infants [12]. Because of that, CYR should be forbidden to sheep producing milk because of these possible toxic effects. The US Environmental Protection Agency (EPA) declared CYR as a potential carcinogenic chemical. The maximum residue limits (MRLs) for CYR was in the range of 1–10 mg/kg [13,14,15]. From all of the above, the importance of monitoring and detecting CYR in food and other environmental matrices become clear and necessary for public health safety.

Currently, there are several analytical techniques reported in literature for detecting CYR, such as gas chromatography–mass spectrometry (GC–MS) [16,17,18,19], high performance chromatography (HPLC) with ultraviolet detection [16,20,21,22], HPLC with mass detection [23,24,25], fluorimetry [26], and capillary electrophoresis [27]. Other techniques are based on enzyme linked immune-sorbent assay [28,29] and vibrational spectrometric procedures [30]. Almost of these reported methods revealed high accuracy and sensitivity but they exhibited many drawbacks combined with their instrumentation and reagents used cost. In addition, they suffer from prolonged sample pretreatment or extraction steps that affect the easiness of their applicability for routine analysis. On the other hand, techniques based on potentiometric transduction have recently attracted much attention because of their simplicity, high reliability, sensitivity, and selectivity towards targeted species [31,32,33,34]. Lately, ion-selective-electrodes (ISEs) incorporating molecularly imprinted polymers (MIP) was a step forward towards organic ions detection [33,34,35,36,37]. Trace-level detection in potentiometric ion-sensors can be achieved at zero-current trans-membrane ion fluxes [38]. This drawback is overcome by using solid-contact potentiometric ISEs (SC/ISEs), in which the inner filling solution was completely removed and the ill-defined interface between the ion-sensing membrane (ISM) and the conductive substrate was eliminated by inserting an electron-to-ion transducing material [39,40,41]. Screen printed solid-contact platforms could be a promising technique for organic ions determination. To the best of our findings, only two cited papers for screen-printed potentiometric sensors based on MIPs was found [37,42].

In this work, we designed and fabricated a novel screen-printed platform integrated with tailor-made MIPs for potentiometric detection of cyromazine pollutant. The solid-contact transducer was polyaniline (PANI). The MIP nano-beads were dispersed in polymeric (PVC) membrane and acted as sensory receptors for the selective recognition of cyromazine. The proposed sensors were successfully applied for the detection of cyromazine in commercial preparations and soil samples.

## 2. Materials and Methods 

### 2.1. Chemicals and Reagents

Cyromazine (98.5%), bispyribac sodium, diquate dibromide, melamine, acetamipride, dinotefuran, imidachloprid, flucarbazone, and atrazine were purchased from Dr. Ehrenstorfer GmbH (Stuttgart, Germany). Polyaniline (emeraldine salt) (Average *M*_w_ > 15,000, 3–100 μm particle size), methacrylic acid (MAA), ethylene glycol dimethacrylate (EGDMA), benzoyl peroxide (BPO), potassium tetrakis (3,5-bis (trifluoromethyl) phenyl) borate (KTFPB), and acetonitrile were purchased from Sigma-Aldrich Inc. (St. Louis, MO, USA). High molecular weight poly(vinyl chloride) (PVC), sodium tetraphenyl borate (NaTPB), dioctyl phthalate (DOP), and tetrahydrofuran (THF) were obtained from Fluka AG (Buchs, Switzerland). Tetrahydrofuran (THF) was freshly distilled prior to use. For pesticide technical formulation, a nominee-kz, 3% soluble liquid (SL) of cyromazine, was purchased from Kafr El*-*Zayat Pesticides and Chemicals Company (Gharbia, Egypt). All other chemical reagents were of analytical grade and used without any further purification. A stock cyromazine solution (10^−2^ M) was prepared by dissolving 0.166 g pure cyromazine in 100 mL distilled water and the pH of the solution was adjusted to pH 3.5 using 0.1 M acetic acid. Dilutions of CYR (10^−3^–10^−7^ M) were prepared in 50 mM acetate solution, pH 3.5.

### 2.2. MIPs Synthesis

Cyromazine MIP beads were tailored using the precipitation polymerization method. Template CYR (1.0 mmol), MAA (3.0 mmol), EGDMA (3.0 mmol), acetonitrile (20 mL), and BPO (70.0 mg) were added to a 25-mL glass-capped bottle successively. Solution homogenization was obtained after sonication for 15 min. The solution is then degassed with N_2_ flow for 10 min to expel all dissolved oxygen dissolved in the solution. For complete polymerization, the reaction bottle was sealed and rotated slowly using a magnet rotor in oil bath for 18 h at 70 °C. The obtained MIP beads were washed with acetic acid/methanol (2:8, *v*/*v*), methanol stepwise by Soxhlet extraction to remove the template. The resulting polymer was left over night at room temperature for complete dryness. Under similar conditions and in absence of cyromazine, the non-imprinted polymer (NIP) was also prepared.

### 2.3. Planar Electrode Development

**Figure 1:**
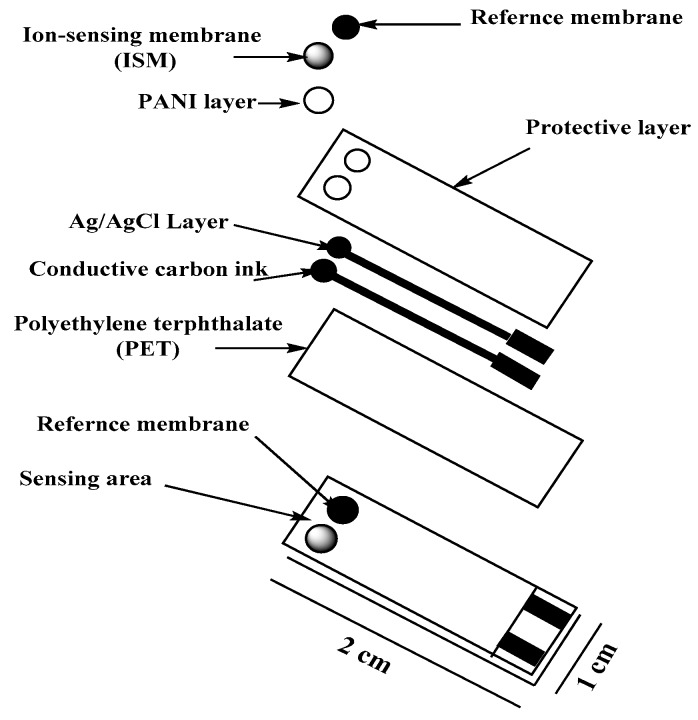
Scheme of the different layers, and final design of the potentiometric strip cell.

The design of the ceramic screen-printed electrode (SPE) is shown in Figure 1. It contains two screens; one is made from carbon and the other from Ag/AgCl. The two screens were printed on alumina substrate of 0.1 mm thickness and 35 mm length. The screen for either carbon or Ag/AgCl ink printing was of 2 mm width. PANI was dissolved in THF (1 mg/mL), and 10 μL was deposited by drop-casting onto the carbon sensing area. After drop-casting, the solution was left to dry for 3 min. The CYR-selective membrane contained 100 mg of the components in 1.5 mL THF as: MIP or NIP beads (10 %), KTFPB (1.5%), PVC (30.5%), and DOP (58%). The reference membrane cocktail was prepared by dissolving 78.1 mg polyvinyl butyral (PVB), 50 mg NaCl in 1 mL methanol. After membrane preparation, a 15 µL of the two different membrane cocktails were drop-casting onto the SPE and reference membrane, respectively and left to dry overnight.

### 2.4. Potentiometric Measurements 

The electromotive forces (*emf*) were measured at 25 ± 1 °C with an Orion (720/SA pH/mV meter, Cambridge, MA, USA). Potentiometric measurements were performed by immersing the designed potentiometric cell in stirred solutions. A correction was made for the EMF values according to the Henderson equation to eliminate the liquid-junction potential. Activity coefficients of the working standard solutions of CYR were calculated by the Debye–Huckel approximation. The potentiometric performance characteristics were calculated following the IUPAC recommendations [43,44]. All experiments were performed with at least three electrodes.

### 2.5. Constant-Current Chronopotentiometry

Chronopotentiometric measurements were carried out to test short-term potential stability. It was performed using Metrohompotentiostat/galvanostat (Autolab, model 204) purchased from Metrohom Instruments (Herisau, Switzerland). The electrodes under study had an area of 2.0 mm^2^ and were connected as the working electrode in a one-compartment cell in 0.01 M CYR at room temperature. The reference electrode was an Ag/AgCl/KCl (3 M) single junction (Model 6.0733.100, Metrohm, Zurich, Swiss), and the auxiliary electrode was a Pt wire. A constant current of ±1 nA, applied to the working electrode for 60 s followed by a reversed current for another 60 s.

### 2.6. Applications to Real Samples

The designed ISEs were introduced to test their applicability in detecting the concentration of CYR inside real samples. The samples containing CYR were commercial soluble liquid (SL) formulation, soil and agricultural waste water samples collected from different agricultural lands sprayed with cyromazine pesticide. A locally present herbicide (Nominee-kz, 3% soluble liquid (SL) of cyromazine) was purchased from Kafr El*-*Zayat Pesticides and Chemicals Company (Gharbia, Egypt). Accurately 0.5–1.0 mL of the formulation were transferred to 100-mL measuring flask and diluted to the mark by 50 mM acetate solution at pH 3.5. The potential readings of the test solutions were recorded and compared with a calibration plot prepared from (10^−2^ to 10^−6^ M) standard CYR solutions under the same conditions of measurements. 

A 250 g of different soil samples were collected from agricultural lands, soaked into 250 mL water and sonicated for 2 h. After filtration, the obtained filtrate was then analyzed by the proposed potentiometric cell and the results were compared to that obtained by the official HPLC standard method [45].

## 3. Results and Discussions

### 3.1. Characterization of MIP Particles

**Figure 2:**
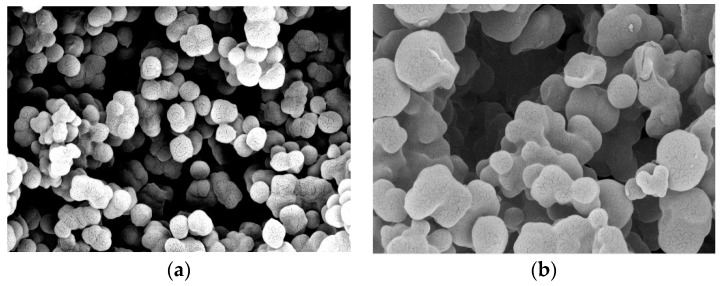
SEM images of (**a**) MIP and (**b**) NIP beads.

The imprinted polymer was prepared by the precipitation polymerization method. The yields for MIPs and NIPs were 53.8%, and 56.4%, respectively. The scanning electron microscope (SEM) was used for characterizing the morphology of the particles. As shown in Figure 2, the observed discrete polymer microspheres were in the low micrometer size range with narrow particle size distributions. The number-average diameter (*D*_n_) of both NIPs and MIPs was 2.58 and 2.78 µm, respectively. These uniform-sized beads give a good dispersion in the polymeric ISE membrane, reduce the membrane resistance, and induce more binding sites available in the membrane. 

**Figure 3:**
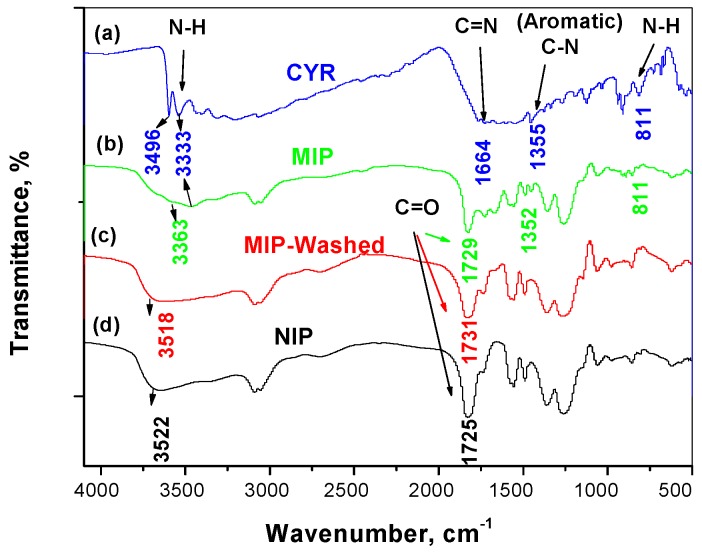
FT-IR spectra for: (**a**) CYR; (**b**) DCP/MIP, (**c**) MIP/washed and (**d**) NIP beads.

Characterization of the resulting polymer was carried out by FTIR spectral analysis. As shown in Figure 3a, the spectrum of CYR showed stretching N–H peaks at 3496 and 3333 cm^−1^. The peaks were strong and of medium intensity. In addition, broad and strong peaks around 1664 and 1355 cm^−1^ assigned to stretching –C=N– and –C–N stretching vibrations. All these peaks were clearly present in the spectrum of MIP/MAA before the removal of CYR (Figure 3b) and completely disappeared in the FT-IR spectrum of the MIP particles after CYR removal (Figure 3c). As shown in Figure 3c,d, they exhibited a strong broad band at 3518 and 3522 cm^−1^ for νOH stretching assigned to –OH group present in the monomer used. This peak appeared in both NIP and washed MIP particles. It is shifted to 3336 cm^−1^ in the spectrum of the un-washed MIP (Figure 3b). This can be attributed to the contribution of –OH group in the monomer in the complexation with the template through hydrogen bond formation. A sharp and strong peak appeared at 1729, 1731, and 1725 cm^−1^ assigned for –C=O group in un-washed MIP washed MIP and NIP particles, respectively. This peak is commonly present in all IR-spectra because of the use of EGDMA cross-linker. From all of the above, we can prove the possibility of the interaction between the N–H group and the carboxylic group of MAA. This confirms the imprinting process of CYR using MAA as a functional monomer.

### 3.2. Characteristics of the Proposed Sensors

**Figure 4:**
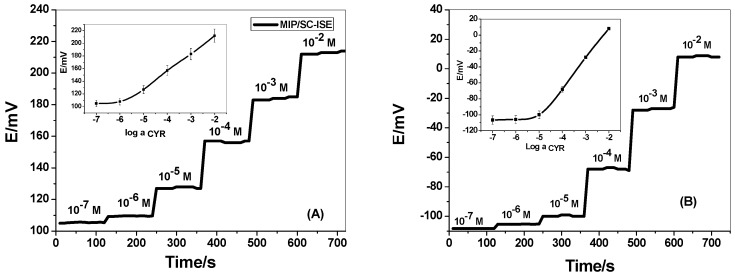
Potentiometric response curves of: (**A**) MIP/PANI-SCISE; and (**B**) TPB/CYR/PANI-SCISE in 50 mM acetate solution of pH 3.5.

The presented new disposable potentiometric strip cell consists of either MIP/PANI or TPB/PANI based CYR-SCISE. The strip cell has been batch produced in the laboratory at a low cost and can easily be transferred to a high throughput, highly parallel, and mass manufacturing process. This would reduce the cost of production and increase reproducibility of the device. In this Technical Note, we report for the first time a new disposable potentiometric strip cell for cyromazine detection. Cyromazine-selective membranes have two sensory materials. These include the synthetic receptor (MIP beads) and TPB/CYR ion association complex. The potential response measured by these sensors was presented in Figure 4. For MIP/PANI-SCISE, it revealed a Nernstian response towards CYR ions with cationic slope of 28.1 ± 2.1 (r^2^ = 0.9999) mV/decade over a linear range 1.0 × 10^−2^–5.2 × 10^−6^ M with a detection limit 2.2 × 10^−6^ M. Membrane sensors based on NIP particles were tested as a control. These sensors showed a worse response performance towards CYR as compared with that contain the MIP beads under the same conditions. This confirms the successful use of these tailored receptors as sensory elements in potentiometric transduction. For TPB/CYR/PANI-SCISE, it exhibited a potentiometric response towards CYR ions with Nernstian slopes of 36.4 ± 1.6 (r^2^ = 0.9991) mV/decade over the linear concentration range 1.0 × 10^−2^–5.7 × 10^−5^ M with detection limit of 8.1 × 10^−6^ M. All potentiometric characteristics of the presented sensors were presented in Table 1.

The ability of the screen-printed platforms to resist long-term storage was examined. The sensors were pre-conditioned in 10^−5^ M CYR for 1 h followed by pre-conditioning in 10^−2^ M NaCl for another 2 h. In preliminary tests, the sensors were stored in a dry place and their performances were evaluated after a given time. No significant changes in their performances were observed after re-calibrating these sensors everyday over one month. This revealed that the sensors can be used dried and stored for one month. Re-calibrating the sensors for longer periods of time (i.e., 8 weeks) and the analytical features of these sensors were recorded. It has been noticed that after 8 weeks of daily use, the limit of detection increased up to 3 × 10^−5^ M, and the potentiometric response declined and the sensitivity decreased. From all of the above, the potentiometric strip cell can be stored in dry conditions for prolonged periods with no significant loss of performance characteristics.

Effect of pH on the potential response of the proposed ISEs was tested. The potential-pH relations revealed no potential variation by more than that ± 1 mV within the pH range of 3.0–4.5. At pH < 3, the potential response begins to decline. This can be attributed to the formation of the trivalent ion of CYR. At pH > 4.5, the potential begins to decline again due to the formation of neutral CYR species. The pka of CYR was reported to be 5.52 [46]. From all of the above, 50 mM acetate buffer background of pH 3.5 was chosen for all subsequent measurements.

The selectivity coefficient (*K^Pot^_i__,_* *_j_)* determines the ability of an ISE to respond to an analyte *I* against an interfering ion *j.* The selectivity coefficients of the CYR electrodes towards foreign compounds were calculated using the modified separate solution method (MSSM) [47]. As these values are small they are tabulated as the negative logarithm (Table 2). The small selectivity coefficient values reflect the high selectivity of the proposed electrodes towards CYR against the studied interfering ions. Evidently, MIP/PANI-SCISE exhibited excellent selectivity towards CYR over melamine, atrazine, Na^+^, K^+^, Cu^2+^, and Fe^3+^ ions. On the other hand, TPB/CYR/PANI-SCISE revealed excellent selectivity towards CRY over diaquat, acetamipride, dinotefuran, imidachloprid, bispyribac, and flucarbazone ions.

From all of the above, the results obtained reflect excellent selectivity for the proposed sensors and offer a great potential for trace-level monitoring of CYR in environmental samples as a low cost, disposable alternative for those applications where conventional sensors are not affordable.

### 3.3. Potential Stability and Conditioning Time

**Figure 5:**
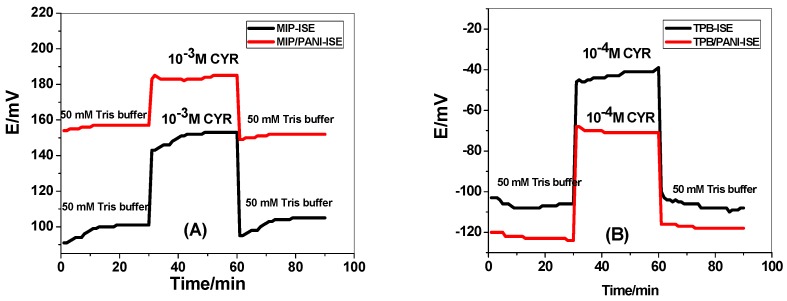
Water-layer tests for the CYR-ISE (**A**) MIP and (**B**) TPB, with/without PANI solid contact.

Conditioning time effect on the long-term potential stability and potential response of the proposed electrodes is shown in Figure 5. The effect of conditioning time was tested by inserting freshly prepared ISEs in 10^−3^ M CYR in acetate solution pH 3.5, and then the potential was monitored for 60.0 min. A potential drift of 115.2 and 190 *μ*V/s was observed for MIP/ISE and TPB/ISE, respectively. The potential stability was enhanced and the drift was lowered to 23.2 and 28.3 *μ*V/s for MIP/ISE and TPB/ISE, respectively. From the results obtained, we can conclude that a water layer was formed between the sensing membrane and conductive substrate in the absence of PANI layer. This is responsible for the observed long-term potential drift. In contrast, the modified ISEs with PANI layer revealed a stable behavior and the equilibrium were reached rapidly after the alteration. This presents evidence for the hydrophobicity of PANI layer through no water layer formation.

**Figure 6:**
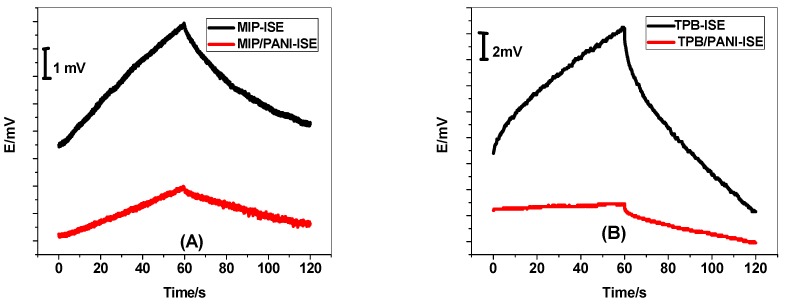
Chronopotentiometry for CYR-ISE (**A**) MIP and (**B**) TPB, with/without PANI solid contact.

Chronopotentiometric technique was used to evaluate the short-term potential stability of the developed PANI nanocomposite [48]. Constant current (±1 nA for 60 s.) was applied on the studied electrodes and the potential response was recorded in 1 × 10^−3^ M CYR in acetate solution pH 3.5. Typical chronopotentiograms of PANI nanocomposite and screen-printed platforms without PANI layer are shown in Figure 6. The total resistance for MIP/PANI-SCISE and TPB/CYR/PANI-SCISE was found to be 0.15 and 0.47 ΩM, respectively. On the other hand, the total resistance for MIP/ISE and TPB/ISE was found to be 0.2 and 1.13 ΩM, respectively. The potential drift for MIP/PANI-SCISE and MIP/ISE was found to be 30.1 and 76.3 *μ*V/s, respectively. The low frequency capacitances for sensors based on MIP beads were 33.2 ± 1.1 and 13.1 ± 0.7 *μ*F, respectively. The potential drift for TPB/PANI-SCISE and TPB/ISE was found to be 35.2 and 175.1 *μ*V/s, respectively. The low frequency capacitance for TPB/PANI-SCISE and TPB/ISE was found to be 28.4 ± 1.2 and 5.7 ± 0.8 *μ*F, respectively.

### 3.4. Analytical Applications 

The proposed ISEs were applied successfully for the assessment of CYR in Nominee-kz, 3% soluble liquid (SL) formulation, and soil samples in triplicate measurements for each sample. The results of pesticide formulation are shown in Table 3. These data were compared with results obtained by measuring cyromazine using the standard method [45].

Different soil samples were collected from different agricultural lands sprayed with cyromazine pesticide. At first, the sensors were calibrated by using the linear equation for cyromazine, and then under the same batch the sensors were used in the sample analysis directly. Similar results are obtained using the official standard method (Table 4). It can be seen the proposed sensors have promising feasibility for determination of cyromazine pesticide in different complex samples.

## 4. Conclusions

In this work, we describe the fabrication and characterization of two different solid contact screen printed electrodes for rapid assay of cyromazine in pesticide formulation and soil samples. Polyaniline (PANI) was used as an ion-to-electron transducing material. This study reinforced the excellent characteristic of PANI as a good solid contact for ISEs to eliminate the water layer formation between the solid contact layer and the membrane sensor. The sensors MIP/PANI-SCISE and TPB/CYR/PANI-SCISE showed Nernstian responses towards CYR ions with slopes of 28.1 ± 2.1 (r^2^ = 0.9999) and 36.4 ± 1.6 (r^2^ = 0.9991) mV/decade over a linear range 1.0 × 10^−2^–5.2 × 10^−6^ and 1.0 × 10^−2^–5.7 × 10^−5^ M with a detection limit 2.2 × 10^−6^ and 8.13 × 10^−6^ M, respectively. The proposed electrodes showed a high selectivity for CYR over common interfering pesticides. The electrodes were used successfully for determination of CYR in soluble liquid (SL) formulation and soil samples with high accuracy and precision.

## Figures and Tables

**Table 1:** Potentiometric characteristics of CYR sensors in 50 mM acetate solution of pH 3.5.

Parameter	MIP/PANI-ISE	NIP/PANI-ISE	TPB/PAN-ISE
Slope (mV/decade)	28.1 ± 2.1	12.7 ± 0.5	36.4 ± 1.6
Correlation coefficient (r^2^)	0.9999	0.9957	0.99916
Detection limit (M)	2.2 × 10^−6^	5.0 × 10^−6^	8.13 × 10^−6^
Linear range (M)	5.2 × 10^−6^	1.0 × 10^−5^	5.7 × 10^−5^
Working pH range (pH)	3–4.5	3–4.5	3.0–4.5
Response time (s)	<10	<10	<10
Accuracy (mV%)	99.2	98.2	99.1
Precision (mV%)	1.1	0.9	1.3

**Table 2:** Potentiometric selectivity coefficients (*log*$k_{CYR,B}^{pot}$) of CYR membrane sensors plasticized with DOP in 50 mM acetate solution of pH 3.5.

Interfering Ion, X	*log K^pot^_M,X_*	
	MIP/PANI-ISE	TPB/PAN-ISE
Diaquat	−2.03	−3.74
Acetamipride	−2.80	−4.63
Dinotefuran	−4.02	−6.07
Imidachloprid	−4.35	−6.44
Bispyribac	−4.26	−6.53
Flucarbazone	−6.01	−7.27
Melamine	−5.32	−4.12
Atrazine	−4.11	−3.91
Na^+^	−7.51	−2.43
K^+^	−6.62	−2.34
Cu^2+^	−8.1	−4.13
Fe^3+^	−9.31	−5.34

**Table 3:** Determination of CYR in commercial pesticide formulation using MIP/PANI-SCISE.

Commercial Product	Label (*w*%/*v*%)	^a^ Found			
		Potentiometry	RSD, %	Official Standard Method [45]	RSD, %
Nomenee-kz, Kafr El*-*Zayat Pesticides and Chemicals Company (Gharbia, Egypt)	3	2.78 ± 0.2	92.6	2.83 ± 0.05	94.3

^a^ Average of five measurements ± standard deviation.

**Table 4:** Determination of cyromazine in some soil samples using MIP/PANI-SCISE.

Sample	Amount of Cyromazine (µg/g)	
	Potentiometry	Official Standard Method [45] ^a^
Sample 1	5.6 ± 0.7	4.7 ± 0.6
Sample 2	8.3 ± 0.3	8.7 ± 0.2
Sample 3	10.2 ± 0.5	9.3 ± 0.4

^a^ Average of five measurements ± standard deviation.
